# Mesenchymal stem cells and extracellular matrix scaffold promote muscle regeneration by synergistically regulating macrophage polarization toward the M2 phenotype

**DOI:** 10.1186/s13287-018-0821-5

**Published:** 2018-04-03

**Authors:** Xinyu Qiu, Shiyu Liu, Hao Zhang, Bin Zhu, Yuting Su, Chenxi Zheng, Rong Tian, Miao Wang, Huijuan Kuang, Xinyi Zhao, Yan Jin

**Affiliations:** 1 State Key Laboratory of Military Stomatology & National Clinical Research Center for Oral Diseases & Shaanxi International Joint Research Center for Oral Diseases, Center for Tissue Engineering, School of Stomatology, Fourth Military Medical University, Xi’an, Shaanxi 710032 China; 2 Research and Development Center for Tissue Engineering, Fourth Military Medical University, Xi’an, Shaanxi 710032 China; 3 Department of Orthodontics, School of Stomatology, Fourth Military Medical University, Xi’an, Shaanxi 710032 China; 4 Department of Stomatology, General Hospital of Tibet Military Region, Lhasa, Tibet 850007 China; 5 State Key Laboratory of Military Stomatology, Department of Dental Materials, Fourth Military Medical University, Xi’an, Shaanxi 710032 China; 6 Department of Dental Materials, School of Stomatology, Fourth Military Medical University, Xi’an, Shaanxi 710032 China

**Keywords:** Mesenchymal stem cells, Macrophage polarization, Extracellular matrix, Tissue regeneration, Volumetric muscle loss

## Abstract

**Background:**

Skeletal muscle plays an important role in the body’s physiology but there are still no effective treatments for volumetric muscle loss (VML) resulting from severe traumatic injury or tumor excision. Recent studies show that a tissue engineering strategy using a compound containing mesenchymal stem cells (MSCs) and decellularized extracellular matrix (ECM) scaffold generates significant regenerative effects on VML injury, but the underlying mechanisms are not fully understood.

**Methods:**

The characteristics of human umbilical cord MSCs, including multiplication capacity and multidifferentiation ability, were determined. We constructed a compound containing MSCs and decellularized ECM scaffold which was used for tissue regeneration in a VML model.

**Results:**

We found that MSCs and decellularized ECM scaffold generated synergistic effects on promoting skeletal muscle tissue regeneration. Interestingly, both MSCs and decellularized ECM scaffold could promote macrophage polarization toward the M2 phenotype and suppress macrophage polarization toward the M1 phenotype, which is widely regarded as an important promoting factor in tissue regeneration. More importantly, MSCs and decellularized ECM scaffold generate synergistic promoting effects on macrophage polarization toward the M2 phenotype, not just an additive effect.

**Conclusions:**

Our findings uncover a previously unknown mechanism that MSCs and decellularized ECM scaffold promote tissue regeneration via collaboratively regulating macrophage polarization.

**Electronic supplementary material:**

The online version of this article (10.1186/s13287-018-0821-5) contains supplementary material, which is available to authorized users.

## Background

Skeletal muscle is one of the most important parts belonging to soft tissue. For traumatic or surgical reasons, volumetric muscle is lost, which is followed by resultant functional deficits [1]. Therapy for volumetric muscle loss (VML) is one of the worldwide medical concerns for doctors and public healthcare systems. For now, the standard treatment for VML injuries is transplantation of muscle flaps from existing host tissue combined with physical rehabilitation. For example, functional muscle has been transplanted in the forearm [2], elbow [3] and lower extremities [4]. However, it is worth noting that muscle flaps cannot fully restore the lost muscle fibers in terms of the physiology and functionality. Besides, these procedures cause large surgical trauma to the patients and require an extraordinary level of surgical expertise.

Under this circumstance, skeletal muscle tissue engineering and regenerative medicine therapies provide hopeful treatment for the loss of a large volume of musculature. Seed cells and extracellular matrix (ECM) scaffolds are widely used in tissue engineering construction, either alone or in combination [5–8]. Especially, combination of mesenchymal stem cells (MSCs) and decellularized ECM was deemed to be the classical strategy to repair tissue injury [9–12]. It has been postulated that MSCs have the ability to self-renew, differentiate into somatic cells and release growth factors to influence the recipient cells, which play an important role in regenerative medicine [13, 14]. Meanwhile, the decellularized ECM is the noncellular part with a network of macromolecules and is arranged in a unique three-dimensional organization, whose composition and structure varies from tissues. It produces a fundamental effect on cell survival, motility and communication, which can promote a constructive skeletal muscle response after experimentally induced skeletal muscle injury in animal models [15–19]. Compared with single use of decellularized ECM scaffold or MSCs, transplantation of a compound combining decellularized ECM scaffold with MSCs had a better therapeutic effect [20–22]. However, the underlying mechanism is still not very clear.

The polarization of macrophages in response to environmental cues has been widely investigated in skeletal muscle [23–25]. According to the reported data, MSCs play an important role in modulating the immune system. Specifically, these cells are able to suppress the immune system through direct interactions as well as releasing numerous bioactive soluble factors [26, 27]. It has been reported that MSCs can directly polarize naïve macrophages toward the M2 phenotype to exert a therapeutic effect on skin, brain and muscle [28–30]. Macrophages are major players in the progression of inflammation as well as tissue regeneration through classic (M1) or alternative (M2) polarization [31]. M1 phenotype cells relate to tissue destruction via secretion of proinflammatory cytokines, such as tumor necrosis factor alpha (TNF-α), interleukin-1β (IL-1β) and interleukin-6 (IL-6). To the contrary, cells with the M2 phenotype promote tissue remodeling by releasing lots of anti-inflammatory cytokines, such as interleukin-10 (IL-10) and interleukin-13 (IL-13). Thus, macrophages can promote both positive and negative outcomes depending on the phenotype to which the macrophage transits [30, 32]. For the decellularized ECM, macrophages can shape the immune microenvironment in traumatic muscle wounds by guiding interleukin-4-dependent macrophage polarization, which induces a proregenerative response and improves muscle tissue regeneration [33]. Although both MSCs and decellularized ECM have immunoregulatory ability, whether a combination of MSCs and decellularized ECM can exert a synergistic effect on immune regulation, especially for macrophage polarization, still needs to be verified.

In the present study, we constructed a compound containing MSCs and decellularized ECM to repair VML injury. Our data provide evidence for the synergistic effects of MSCs and decellularized ECM, which may have new significance for stem cell therapy and regenerative medicine.

## Methods

### Animals

All animals were purchased from the Animal Center of Fourth Military Medical University, Xi’an, China. Twelve-week-old Sprague Dawley (SD) rats were used to establish the VML model. The animal protocols were approved by the Animal Care Committee of Fourth Military Medical University (Approved ID: No. 16096). All animals were housed under specific pathogen-free conditions (24 °C, 12-hour light/12-hour dark cycles and 50% humidity) with free access to food pellets and tap water.

### Cell isolation and culture

Mesenchymal stem cells (MSCs) were isolated from human umbilical cords because they do not require an invasive procedure and do not have the controversies of human embryonic stem cells. The umbilical cords were washed by PBS, and then outer membranes and vessels were isolated and removed. The mechanical dissociation and explanted culture method was used in this experiment. Briefly, the remaining tissues were manually dissected into smaller blocks whose volume was 10 mm^3^ and plated in tissue culture flasks with α-MEM medium (Gibco, USA) supplemented with 10% FBS (Gibco, USA) and 1% penicillin/streptomycin (Invitrogen, USA) for 7 days in a 37 °C, humidified environment with 5% CO_2_. Then, MSCs were detached and passaged with 0.25% trypsin/1 mM EDTA (Gibco, USA) at 80–90% confluence. Passage 5 (P5) cells were identified and used for the experiments of this study.

### Flow cytometric analysis of cell phenotype

Cell phenotypes of cultured MSCs at P5 were detected by flow-cytometric analysis. For identification of the expression of stem cell surface markers, MSCs were harvested and washed by PBS. Then, the single cell suspension was incubated with phycoerythrin (PE)-conjugated human anti-CD29, anti-CD31, anti-CD34, anti-CD44, anti-CD45, anti-CD90 and anti-CD105 (all from eBioscience, USA), respectively. PE-conjugated IgG was used as control. Finally, cells were subjected to flow cytometric analysis with a Beckman Coulter Epics XL cytometer (Beckman Coulter, USA).

### Osteogenic differentiation assay

MSCs were seeded in six-well plates at a density of 1 × 10^5^ cells/well and cultured in a basal medium for 24 hours. Then, the medium was changed to osteogenic medium: α-MEM containing 10% FBS, 1% penicillin/streptomycin, 5 mM β-glycerophosphate (Sigma-Aldrich, USA), 50 μg/ml ascorbic acid (Sigma-Aldrich) and 10 nM dexamethasone (Sigma-Aldrich). The medium was refreshed every 3 days.

For alkaline phosphatase (ALP) staining, after 10 days the medium was discarded, and the samples were washed with PBS twice and fixed with 4% paraformaldehyde (Sigma-Aldrich). ALP staining was performed with a commercial kit (Beyotime, China) according to the manufacturer’s protocol. Cells were cultured for 28 days and the Alizarin red (Sigma-Aldrich) staining was performed according to the manufacturer’s instructions. Photographs were taken by an inverted optical microscope (Olympus, Japan).

### Adipogenic differentiation assay

MSCs were seeded in six-well plates at a density of 1 × 10^5^ cells/well to assess lipid formation in vitro. The medium was replaced by adipogenesis-inducing medium containing 0.5 mM isobutylmethylxanthine (MP, USA), 0.5 mM dexamethasone (MP, USA) and 60 nM indomethacin (MP, USA). After induction for 14 days, Oil Red O (Sigma-Aldrich) staining was performed to determine lipid droplet formation. Photographs were taken by the inverted optical microscope (Olympus, Japan).

### RNA extraction and real-time RT-PCR of mRNA

The total RNA was extracted by Trizol reagent (Invitrogen, USA) according to the manufacturer’s protocol. Then 1000 ng total RNA was reverse transcribed to cDNA using a PrimeScript RT reagent kit (TaKaRa, Japan). Real-time RT-PCR analysis was performed using the SYBR Premix Ex Taq II kit (TaKaRa, Japan) and tested by CFX96TM Real-time RT-PCR System (Bio-Rad, USA). The settings of program were 95 °C for 10 min, 40 cycles of 95 °C for 15 s, and 60 °C for 1 min. β-actin was used as the internal control for quantitation of the target mRNA. The primer sequences for real-time RT-PCR are presented in Table 1.

**Table 1:** Primer sequences

Gene	Forward	Reverse
*IL-10*	5′-CCTCTGGATACAGCTGCGAC-3′	5′-TGAGTGTCACGTAGGCTTCT-3′
*IL-6*	5′-GGACATCGTGTACATCGGCT-3′	5′-CTTCCTTCCCAGCAGGTAGC-3′
*IL-1β*	5′-AGGCTGACAGACCCCAAAAG-3′	5′-CTCCACGGGCAAGACATAGG-3′
*IL-13*	5′-AACAACGTGGAGAAAACCCC-3′	5′-GGGTCGATGGAGTCACATGC-3′
*TNF-α*	5′-GATCGGTCCCAACAAGGAGG-3′	5′-TTTGCTACGACGTGGGCTAC-3′
*IL-4*	5′-GACTCCATGCACCGAGATGT-3′	5′-GTGAGTTCAGACCGCTGACA-3′
*β-actin*	5′-TGGCACCCAGCACAATGAA-3′	5′-CTAAGTCATAGTCGCCTAGAAGCA-3′

### Colony-forming unit fibroblast assays

To assess the colony-forming efficiency of MSCs, single-cell suspensions (P5) with α-MEM containing 10% FBS were seeded in 5-cm-diameter culture dishes (Corning, USA) at a density of 5 × 10^2^ cells/well and cultured at 37 °C in a humidified atmosphere containing 5% CO_2_. The medium was refreshed every 3 days. After culturing for 10 days, the dishes were rinsed with PBS and the cells were fixed by 4% paraformaldehyde (Sigma-Aldrich). The cells were stained with 1% toluidine blue, washed with distilled water and dried for evaluation under the inverted optical microscope (Olympus, Japan).

### Culture of cell aggregate

A total of 2 × 10^5^ MSCs were seeded into a six-well plate with 3 ml basal medium for 3 days. After reaching 90% confluence, the medium was changed to α-MEM containing 100 mg/ml vitamin C (Invitrogen, USA) and 10% FBS for another 14 days. The medium was refreshed every 2 days. Finally, a white membrane structure was observed, and cell aggregate was carefully detached from the culture plates with a cell scraper.

### Morphological analysis of volumetric muscle loss-injured tibialis anterior muscle

TA muscle was isolated from the leg and laid on a platform with the muscle facing away from the platform. At the middle of the proximal and distal thirds of the muscle, the width of the muscle was measured using a slide caliper. The thickness of the middle muscle was also measured by a slide caliper

### Scanning electron microscopy observation

Cell aggregate, native and decellularized heart tissue sections were fixed by 2.5% glutaraldehyde at 4 °C for 12 hours. The samples were anodized in an electrolyte containing 0. 5% wt hydrofluoric acid and 1 M phosphoric acid for 1 hour. After that, the samples were observed by scanning electron microscope (Hitachi, Japan).

### Immunofluorescence staining

Immunofluorescence staining was performed as described previously [34]. Briefly, the sections from TA muscle, native and decellularized ECM were fixed and rinsed. After rinsing, they were permeabilized with 0.03% Triton-X100 for 10 min at room temperature and blocked in 5% BSA at 37 °C for 30 min. The sections were incubated overnight (at least 8 hours) at 4 °C with the primary antibody for iNOS (1:200; Abcam, USA), CD206 (1:200; Abcam), Collagen I (1:500; Abcam), Laminin (1:200; Abcam) and Fibronectin (1:200; Abcam) respectively. After rinsing, the sections were incubated with fluorescence secondary antibody (Cell Signaling, USA) at room temperature for 1 hour. The nuclei were counterstained by Hoechst 33342 (Sigma-Aldrich) for 10 min at room temperature. The results were examined under a confocal microscope (Olympus, Japan). The photographs were evaluated by Image-Pro Plus 6.0 (Media Cybernetics, USA) from three randomly selected views of each specimen.

### Histological and immunohistochemical staining

The harvested cell aggregate, native and decellularized tissue sections from porcine heart and TA muscle from rat were fixed in 4% phosphate-buffered paraformaldehyde for 24 hours, embedded in paraffin. Eight-micrometer-thick serial sections were cut from the paraffin-embedded blocks and underwent H&E staining and Masson’s Trichrome staining. Immunohistochemical staining was performed using standard procedures as described previously [35, 36]. Briefly, sections were incubated with primary antibodies as follows: anti-Col-I (1:200; Santa Cruz, USA), anti-fibronectin (1:200; Abcam) and anti-integrin-β1 (1:200; Abcam). The same source IgG was used for the negative control instead of the primary antibodies. Biotinylated secondary antibodies (1:1000) were purchased from Sigma-Aldrich. The stained sections were observed using the light microscope (Nikon, Japan). The photographs were evaluated by Image-Pro Plus 6.0 (Media Cybernetics, USA) from three randomly selected views of each specimen.

### Tissue extracellular matrix selection and decellularization

Tissue ECM selection was carried out as described previously with minor modification [33]. Briefly, the heart from a 6-month-old pig was selected from the nearest slaughterhouse and was processed following a standard protocol. According to previous studies [33, 37], decellularized cardiac tissue can increase the M2-associated genes compared with other tissues and successfully regenerate the skeletal muscle. Tissues were cut into particle sizes of about 1 mm^3^ and rinsed with distilled water until the blood was cleared. The heart tissues were firstly incubated in 4% peracetic acid (Sigma-Aldrich) on a shaker at 37 °C for 8 hours. Then, tissues were washed with PBS at least three times. Following this, they were transferred to 1% SDS in PBS solution for 48 hours followed by 1% Triton-X100 (Sigma-Aldrich) + 2 mM sodium EDTA (Sigma-Aldrich) solution for 24 hours, changing the solution every 12 hours. Tissues were rinsed with PBS until bubbles were cleared. Finally, processed tissues were incubated in 500 U/ml DNase I (Roche Diagnostics, USA) + 10% Antifungal-Antimycotic (Gibco, USA) in PBS solution for 24 hours. The tissues were washed by PBS and then frozen at -80℃ and lyophilized for 3 days. Lyophilized ECM was crushed into powder using a mortar and pestle with the help of liquid nitrogen. ECM powder was stored at −80 °C and UV sterilized 4 hours prior to use.

### Surgical procedures

Surgical procedures of VML injury were performed in the TA muscle as reported previously [38]. Briefly, a longitudinal skin incision was made along the lateral side of the lower leg using a scalpel. The skin was separated from the fascia by blunt dissection. The fascia covering the TA muscle was then bluntly but gently separated. TA muscle was excised approximately 20% in the VML injury model and the TA muscle weight at the middle third of the muscle. The defect dimensions following spatula removal approximated 10 mm × 7 mm × 3 mm (length × width × depth). Repair of the TA muscle was performed by folding heart decellularized ECM powder, cell aggregate or the compound on the defect area. Prolene (6–0) markers were placed at the corners and margins of the defect to track the area of the defect at the time of harvest. The fascia and skin were closed using vicryl (6–0) and prolene (6–0) interrupted sutures. Then, 100 μl PBS was injected into the defect area as control. Finally, a compression bandage was wrapped around the lower leg for 10 min (Additional file 1). After 2 weeks and 8 weeks, the rats were sacrificed, and their entire TA muscle and inguinal lymph nodes were removed. Inguinal lymph nodes and whole muscle samples for RNA isolation were flash frozen in liquid nitrogen and stored at −80 °C until RNA extraction.

### Biochemical characterization of decellularized ECM

To identify the extent of decellularization, the components of extracellular matrix like collagen (COL) and glycosaminoglycans (GAGs) were measured, and the residual DNA was also evaluated. For DNA evaluation, the decellularized ECM powder was digested in 500 μl papain solution (125 μg/ml papain in 0.1 M PBS with 5 mM sodium EDTA and 5 mM cysteine–HCl at pH 6.0) for 24 hours at 60 °C. Native heart tissues of similar weight were also digested in the same condition as controls. The DNA content was determined using Hoechst 33258 assay [39]. A fluorescence spectrophotometer (excitation wavelength 360 nm, emission wavelength 450 nm; HITACHI, Japan) was used for assessing the amount of resident DNA of decellularized ECM and native tissue. The calf thymus DNA was used for generating a standard curve to quantify the DNA in samples. The GAG content was evaluated via quantifying the amount of glycosaminoglycans using 1,9-dimethylmethylene blue solution [40]. Chondroitin sulfate A was used for generating a standard curve to estimate the sulfated glycosaminoglycans in samples. The absorbance was measured with a microplate reader at a wavelength of 490 nm. The COL content was evaluated via a conventional hydroxyproline assay [41]. The absorbance was measured with a microplate reader at a wavelength of 550 nm and the standard curve was made by hydroxyproline. For histological evaluation, the native heart tissue and skeletal muscle tissue were frozen by isopentane with the help of liquid N_2_, the decellularized tissues were fixed in 4% paraformaldehyde and washed several times with PBS. Then, both native and decellularized tissues were embedded in OCT compound and the samples were sectioned at 10 μm with a cryotome (Leica CM1850 Cryostat, Germany).

### Mechanical function study

The entire TA muscle was isolated from the tendon of rat under anesthesia and quickly transferred into a biological box containing saline buffer solution at 25 °C. A 4–0 silk suture was used to attach the one tendon to the force transducer (BL-420F; Chaoyang Instruments, China) while the other tendon was attached to a hook at the bottom of the biological box. Electrical stimulation (ES) (20 V at electrodes, 0.1 ms square pulse, 600 ms train) was applied to the muscle using electrodes. After adjusting the length of muscle by rotating the measuring head, the optimal length was identified based on the twitch response. The peak isometric contractile force was measured at optimal length from 1 to 150 Hz. An electrical stimulator and the recording system were provided by the biological detector (BL-420F; Chaoyang Instruments, China).

### Statistical analysis

All of the results are representative of data generated in three independent experiments. All numerical values were expressed as the mean ± SD. Comparisons of two groups were done with two-tailed Student’s *t* tests and comparisons of multiple groups were done with ANOVA using the Statistical Program for Social Science. The effect of interaction between MSCs and ECM scaffold was done with factorial-design ANOVA. *P* < 0.05 was considered statistically significant.

## Results

### Preparation of decellularized ECM from porcine heart

**Fig. 1:**
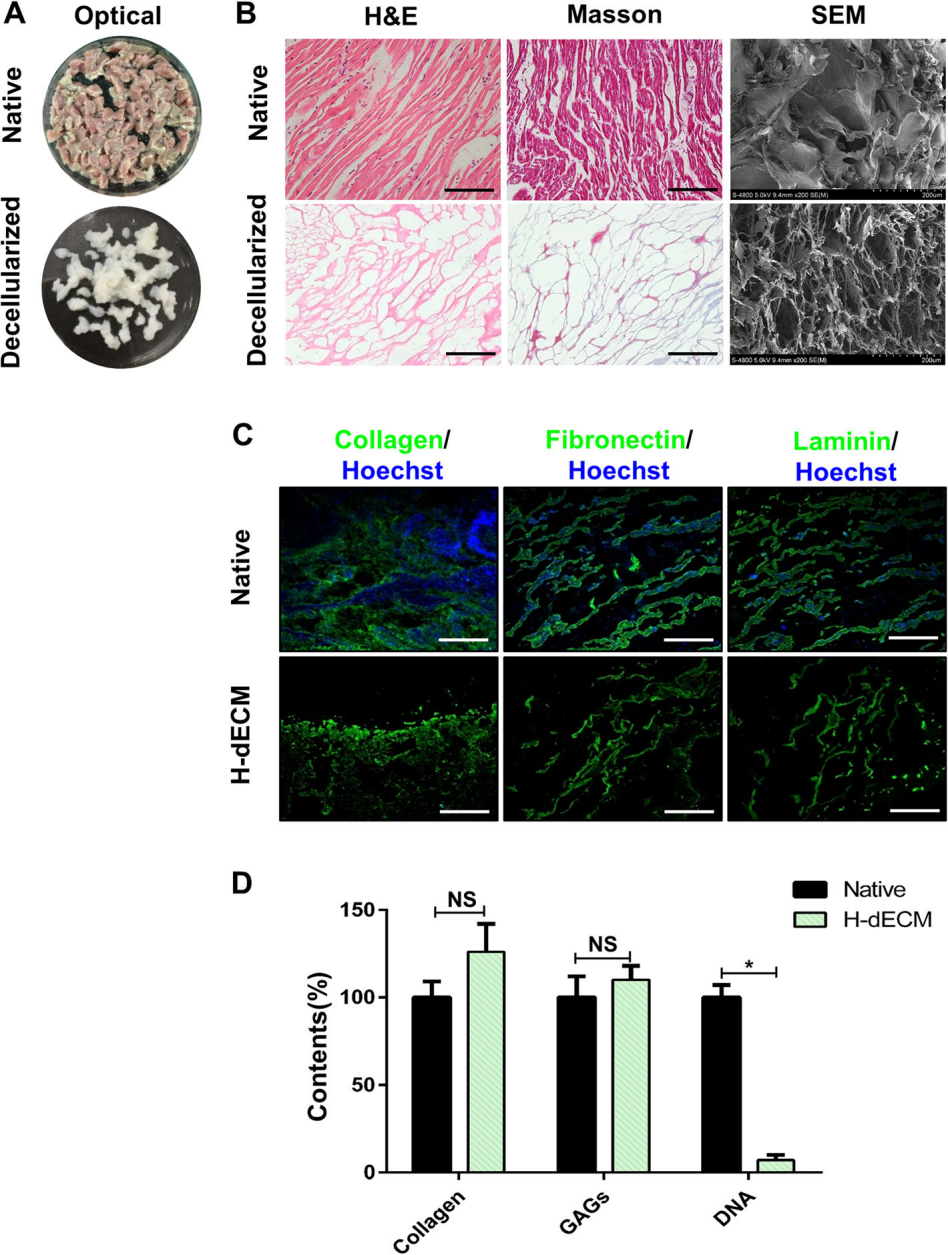
Decellularization of native heart tissue and biochemical analysis. **a** Representative optical images of native and decellularized heart tissue. **b** H&E staining images, Masson’s Trichrome staining images and SEM images of native and decellularized heart tissue. Scale bar, 100 μm. **c** Representative images of immunofluorescence staining for collagen I, fibronectin and laminin of native and decellularized heart tissue. Scale bar, 200 μm. **d** ECM components (COL and GAGs) and DNA contents of native and decellularized heart tissue. All experiments performed in triplicate. Data shown as mean ± SD. *P < 0.05; NS, not significant (*P* > 0.05). H&E hematoxylin and eosin, SEM scanning electron microscopy, H-dECM heart decellularized extracellular matrix, GAG glycosaminoglycan

As the initial step, decellularization of the ECM materials aims to maximize the removal of cellular material while minimizing ECM damage and loss [42]. To obtain the decellularized heart tissue, we used the combination of physical, chemical and enzymatic process methods as described previously with slight modification [33]. Efficiency of decellularization was evaluated in several different ways. H&E staining, Masson’s Trichrome staining and SEM confirmed the absence of cells and cell debris in the matrix after decellularization (Fig. 1a, b). At the same time, we evaluated the preservation of collagen I, fibronectin and laminin in decellularized extracellular matrix by immunofluorescence (Fig. 1c). DNA quantification assay, which was performed before and after decellularization, showed almost full clearance of the cellular contents but good preservation of ECM components like COL and GAGs (Fig. 1d).

### Isolation and characterization of MSCs

**Fig. 2:**
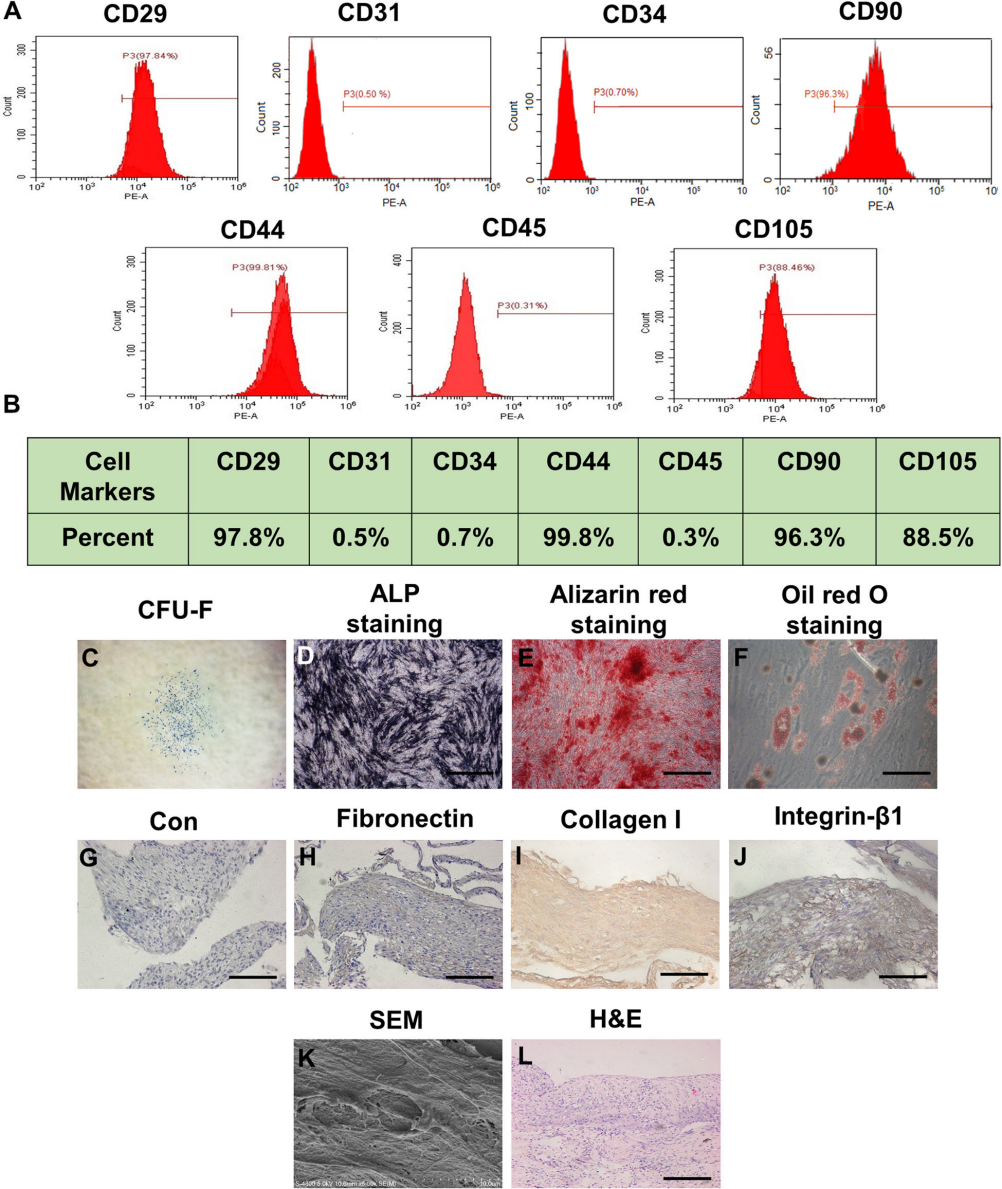
Characterization of human umbilical cord mesenchymal stem cells (hUCMSCs). **a** Flow cytometric analysis of ex-vivo-expanded hUCMSCs revealed positive expression of CD29, CD44, CD90 and CD105, and negative expression of CD31, CD34 and CD45. **b** Representative percentage of cell markers for identification. **c** Representative proliferation of single clone of hUCMSCs. **d** hUCMSCs seeded in plates induced with osteogenic medium for 10 days. Activity of ALP detected by ALP staining. **e** hUCMSCs cultured in osteogenic inductive conditions for 28 days, mineralized nodules found by Alizarin Red staining. **f** Cultured hUCMSCs formed Oil Red O-positive lipid cluster following 14 days of adipogenic induction. **g**–**j** Cell aggregate (**g** control) positively expressed fibronectin (**h**), collagen I (**i**) and integrin-β1 (**j**). **k** Arrangement of cells in cell aggregate observed by SEM. **l** H&E staining showing cell aggregate was dense and contained plenty of cells. *n* = 6 per group. Scale bar, 100 μm. PE phycoerythrin, CFU-F fibroblastic colony-forming unit, ALP alkaline phosphatase, Con control, SEM scanning electron microscopy, H&E hematoxylin and eosin

The purified MSCs were successfully obtained from umbilical cords from donors. They were propagated on a standard plastic dish in vitro and exhibited fibroblast-like morphology. The cells exhibited the characteristic pattern of MSC surface markers, including CD29, CD44, CD90 and CD105, whereas the hematopoietic markers CD31, CD34 and CD45 were negative (Fig. 2a, b). When the MSCs were cultured at a low density, they formed adherent clonogenic cell clusters (Fig. 2c). To investigate the differentiation potential of the MSCs, they were cultured in osteogenic differentiation medium. ALP staining was performed after 10-day induction (Fig. 2d) and mineralized nodules were stained with Alizarin red after 28-day induction (Fig. 2e). After culturing in adipogenesis-inducing medium for 14 days, the MSCs were found to form lipid droplets, as confirmed by Oil Red staining (Fig. 2f). After 14-day induction using the medium containing Vc, MSCs formed the complete cell aggregate which could be detached at the edge of the dishes. Through immunohistochemical staining, we verified that the cell aggregate contained plenty of col-I, fibronectin and integrin-β1 which are important ingredients for cell attachment (Fig. 2g–j). Meanwhile, the results of SEM and H&E staining revealed that the aggregate had many layers of cells and retained much ECM secreted from MSCs (Fig. 2k, l).

### Effects of MSCs and ECM on muscle regeneration and mechanical function recovery post implantation

**Fig. 3:**
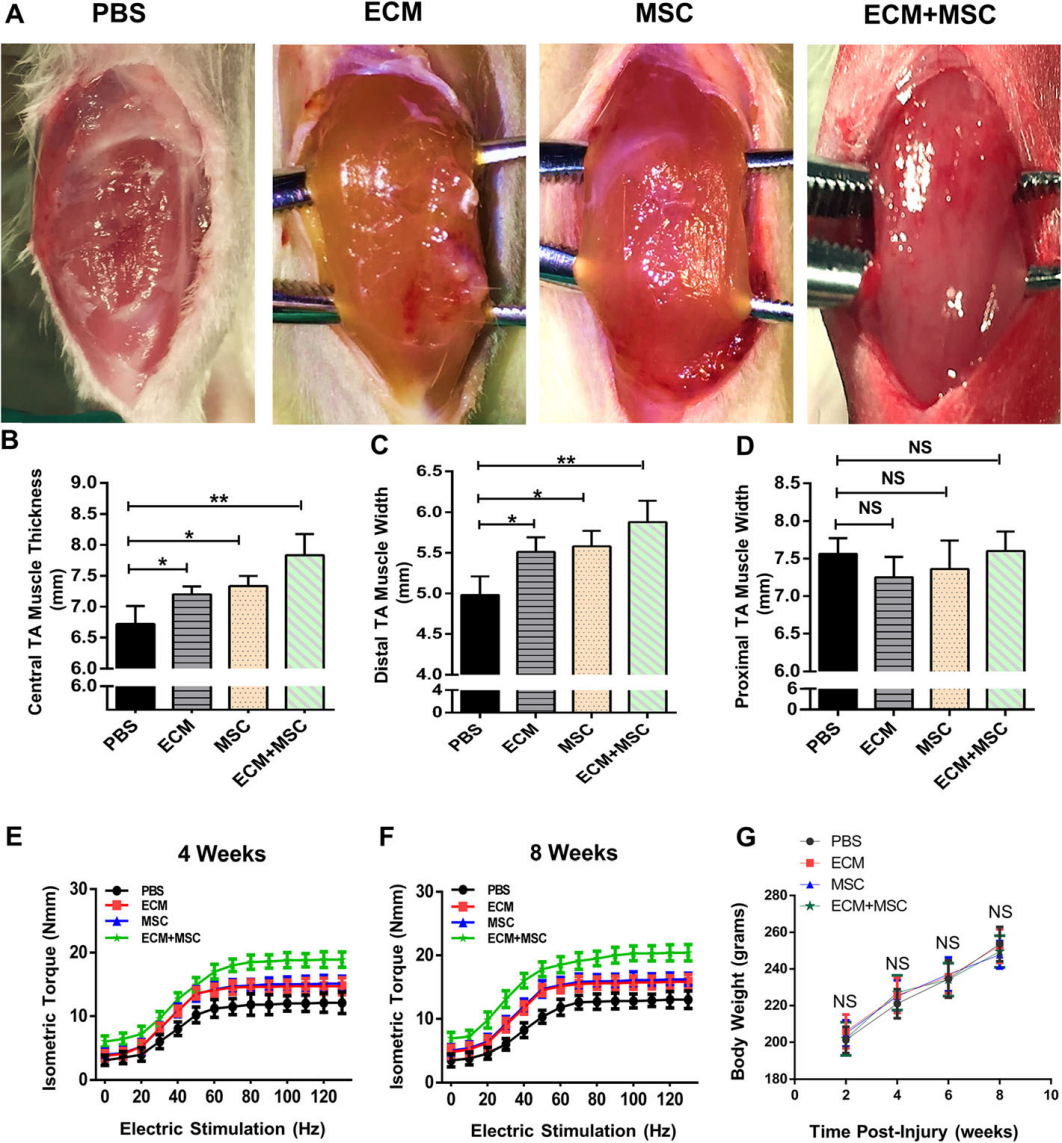
Gross morphology and functional evaluation of TA muscle after VML injury and repair. **a** Gross appearance of VML-injured muscle in four groups. **b–d** Representative central thickness of TA muscle (**b**) and widths of the distal (**c**) and proximal (**d**) thirds of the TA muscle in each group. ECM-treated group, MSC-treated group and compound-treated group can all partially recover central thickness and distal width of TA muscle. **e**, **f** Mechanical function evaluation by ES-induced contractions observed in each group presented at 1 month (**e**) and 2 months (**f**). **g** Mean body weight of four groups over the course of 8 weeks. No differences observed among all groups at each time point. *n*= 6 per group. Data shown as mean ± SD. *P < 0.05; **P < 0.01; NS, not significant (*P* > 0.05). PBS phosphate buffer saline, ECM extracellular matrix, MSC mesenchymal stem cell, TA tibialis anterior

After successfully isolating the MSCs from human umbilical cord and decellularizing the ECM from porcine heart we next constructed the compound containing MSCs and ECM. In order to examine the effect of the compound on the skeletal muscle regeneration, we established the VML model in rat. After injury, MSCs, ECM and the compound were used separately to repair the defect area (Additional file 1). Eight weeks later, we surprisingly found that the void was filled by new muscle and the surface gloss of muscle was also restored in the compound-treated group. Although MSCs or ECM alone can partially attenuate the atrophic appearance of the muscle, the regeneration effect was not comparable with the compound-treated group. As in the PBS-treated group, a longitudinal fissure could still be observed where the defect area was created by surgery (Fig. 3a). Consistent with this observation, compared to the PBS-treated group, all of the other three groups demonstrated improvement of the central thickness and distal width of the TA muscle (Fig. 3b, c) but not for the proximal width (Fig. 3d). In the injured muscles, there is no severe atrophy in the proximal third of tissue compared to the distal muscles. The compound-treated group was observed to have the most obvious regenerative effect among all groups. To evaluate the recovery of mechanical function, ES-induced contractile responses were obtained from all four groups. Force and frequency relationships were summarized at 1 month (Fig. 3e) and 2 months (Fig. 3f). Consistent with the gross morphological observations, the force responses revealed the mechanical function recovery of a different degree after 2 months of implantation compared to the PBS-treated group. As we can see, the compound-treated group had more positive effects on mechanical function recovery than the MSC-treated group or ECM-treated group. Meanwhile, the body weight gain was similar among all experimental groups during the study (Fig. 3g). In general, we found that application of the compound containing MSCs and ECM scaffold could significantly promote the regeneration of muscle fibers and mechanical function recovery after VML injury.

### Histological evaluation of repaired TA muscle

**Fig. 4:**
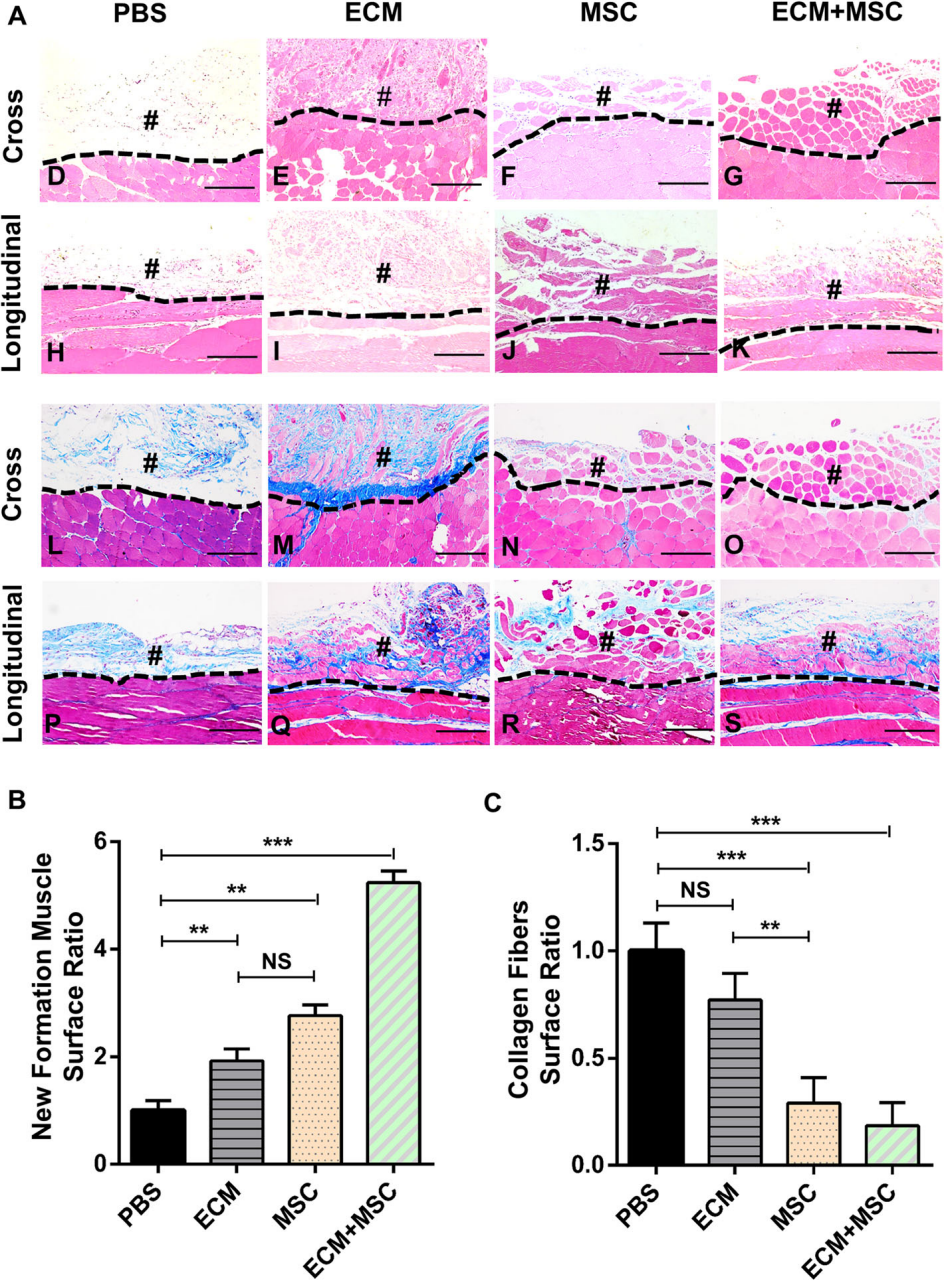
TA muscle histological study following repair in four groups after transplantation for 8 weeks. **a** TA muscles harvested 8 weeks after VML injury. Longitudinal and cross-sections from four groups stained with H&E (D–K) and Masson’s Trichrome (L–S). **b**, **c** Surface ratio of new muscle fibers (**b**) and collagen fibers (**c**) formed in each group analyzed by Image-Pro Plus 6.0. *n* = 6 per group. #Area of the defect. Dashed line represents separation between two areas. Scale bar, 200 μm. Data shown as mean ± SD. **P < 0.01; ***P < 0.001; NS, not significant (*P* > 0.05). PBS phosphate buffer saline, ECM extracellular matrix, MSC mesenchymal stem cell

In order to investigate the changes of TA muscle at histological level after 8 weeks post injury, we stained the sections from TA muscle by H&E and Masson’s Trichrome assay. Obviously, the compound-treated group had the best regeneration effect among all groups, marked by the formation of a band of regenerated muscle fibers in close proximity to the remaining muscle mass. Moreover, few unordered collagen fibers were observed in the defect area (Fig. 4a G, K, O, S). On the contrary, in the ECM-treated group (Fig. 4a E, I, M, Q) and the MSC-treated group (Fig. 4a F, J, N, R), there were few regenerated muscle fibers. As in the PBS-treated group, fibrotic scarring was observed at the defect area with a large accumulation of disarrayed and irregular collagen fibers while no regenerated muscle fibers could be seen (Fig. 4a D, H, L, P). According to the statistical results, the compound-treated group exerted better effect than the other groups both on regeneration of new muscle fibers and reduction of disordered collagen fibers. Meanwhile, the MSC-treated group seems to have had a better effect on inhibiting disordered collagen fibers than the ECM-treated group (Fig. 4b, c). In short, through histological observation, we further confirmed that the compound-treated group had the best regeneration effect among all of the groups.

### Effect of MSCs and ECM on macrophage polarization during muscle regeneration

**Fig. 5:**
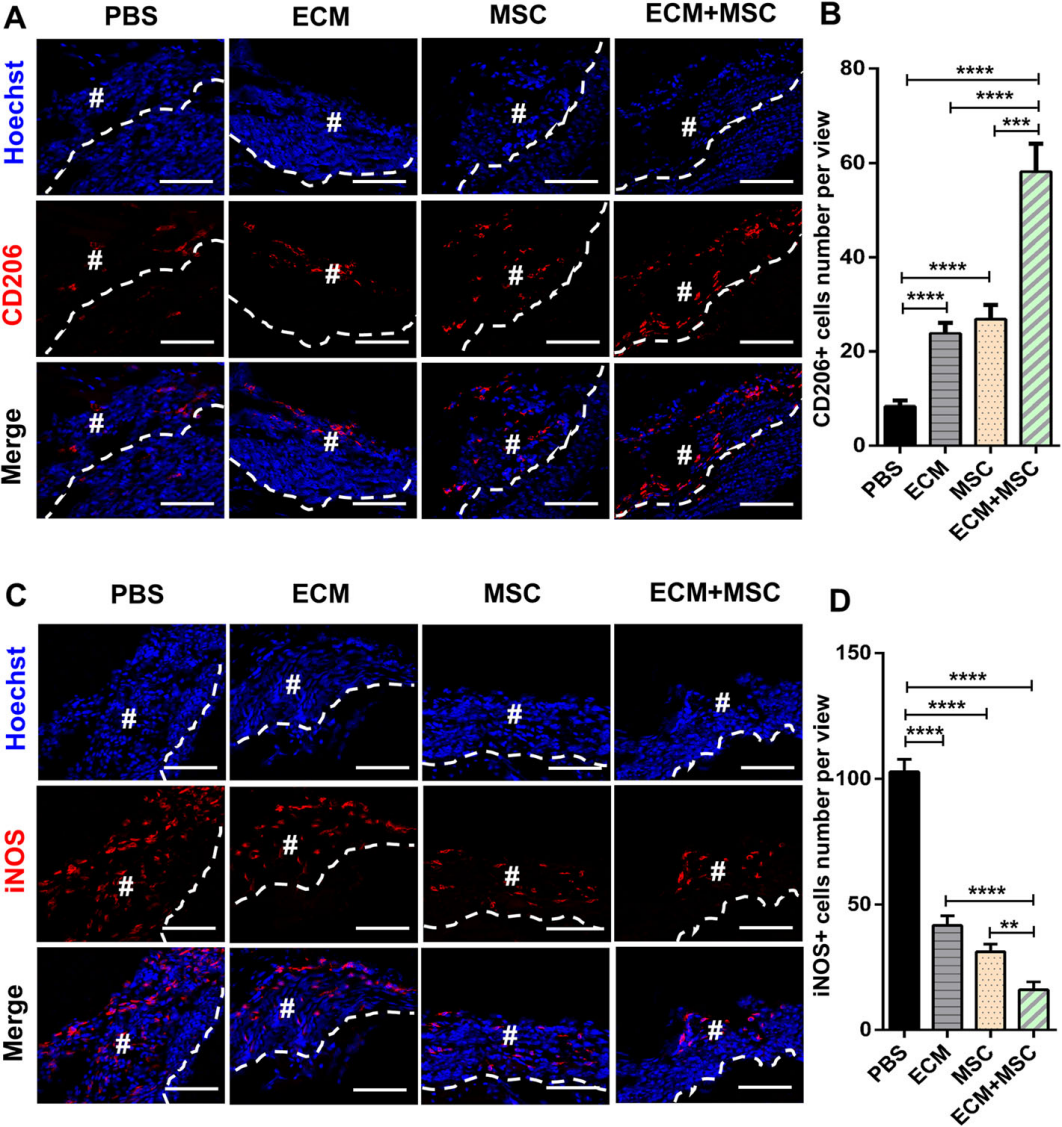
Macrophage polarization toward M2 phenotype after transplantation for 2 weeks. **a** Only a small amount of CD206 (red)-positive macrophage appeared in the PBS-treated group, few in the ECM-treated group and MSC-treated group, and plenty of CD206-positive macrophages detected in the compound treated group. Nuclei stained with Hoechst 33342 (blue). **b** Field of view for CD206-positive macrophages in each group analyzed by Image-Pro Plus 6.0. **c** Plenty of iNOS (red)-positive macrophages showed in the PBS-treated group, meanwhile, only few can be detected in the compound-treated group. Nuclei were stained with Hoechst 33342 (blue). **d** Field of view for iNOS-positive macrophages in each group analyzed by Image-Pro Plus 6.0. *n* = 6 per group. #Area of defect. Dashed line represents separation between two areas. Scale bar, 200 μm. Data shown as mean ± SD. **P < 0.01; ***P < 0.001; ****P < 0.0001; NS, not significant (*P* > 0.05). PBS phosphate buffer saline, ECM extracellular matrix, MSC mesenchymal stem cell, iNOS inducible nitric oxide synthase

To further explore the reason why MSCs and ECM had a therapeutic effect on promoting muscle regeneration, we focus our mind on macrophage polarization. As we all know, the regenerative outcome of MSCs and ECM in animals is correlated with an immunoregulatory M2 macrophage phenotype during remodeling. Both MSCs and ECM could promote macrophage polarization toward the M2 phenotype which exerted positive effects on wound healing [43–45]. We thus hypothesized that application of the compound containing MSCs and ECM could produce a synergistic effect on macrophage polarization. After 2 weeks post injury, the compound-treated group showed the largest population of CD206-positive macrophages which indicated the M2 phenotype in the injury site among all of the groups, while the PBS-treated group had the least (Fig. 5a). No significant difference existed between the ECM-treated group and the MSC-treated group, both of which had more CD206-positive macrophages than the PBS-treated group but fewer than the compound-treated group (Fig. 5b). Besides, the M1 phenotype marked by iNOS-positive macrophages showed the opposite trend compared to CD206-positive macrophages (Fig. 5c, d).

**Fig. 6:**
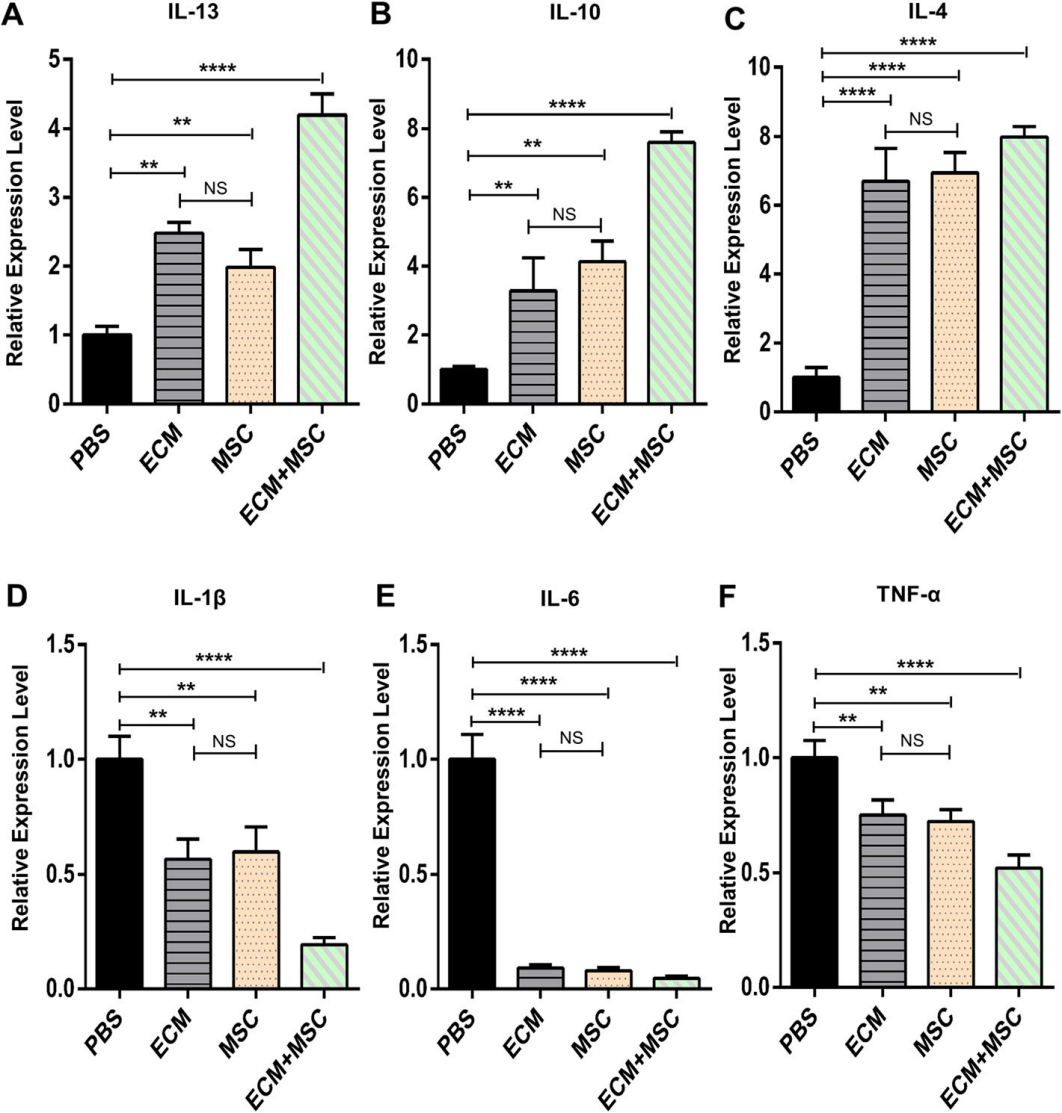
Gene expression in inguinal lymph node after 2 weeks post operation. Gene expression measured in local (inguinal) lymph nodes at 2 weeks after transplantation to measure M1 phenotype-related (TNF-α, IL-1β, IL-6) (**a**–**c**) and M2 phenotype-related (IL-4, IL-10, IL-13) (**d**–**f**) cytokine changes dependent on ECM and MSCs application. *n* = 6 per group. Data shown as mean ± SD. **P < 0.01; ****P < 0.0001; NS, not significant (*P* > 0.05). IL, interleukin, PBS phosphate buffer saline, ECM extracellular matrix, MSC mesenchymal stem cell, TNF-α tumor necrosis factor alpha

Besides, to confirm the expression level of inflammatory factors, we took the inguinal lymph nodes for the real-time RT-PCR test. The results showed that the expression of IL-13, IL-10 and IL-4 which were related to macrophage polarization toward the M2 phenotype was significantly upregulated in all experiment groups compared to the PBS-treated group, especially in the compound-treated group (Fig. 6a–c). On the contrary, the expression of TNF-α, IL-6 and IL-1β which were related to macrophage polarization toward the M1 phenotype was robustly downregulated in the three experiment groups but not in the PBS-treated group (Fig. 6d–f). Taken together, the results suggested that the combination of ECM and MSCs exerted a strong synergistic effect on macrophage polarization to promote skeletal muscle regeneration in the rat VML model. The data was analyzed by using factorial-design ANOVA and showed a synergistic effect between the MSCs and ECM on macrophage polarization toward the M2 phenotype, not just an additive effect.

## Discussion

Over the past decades, numerous studies have focused on developing therapeutic strategies to generate a volume of skeletal muscle for restoring traumatized skeletal muscle. Hence, a variety of tissue engineering approaches have been applied in promoting partial regeneration of lost skeletal muscle fibers at the site of VML. MSCs and ECM scaffold have been widely used in tissue engineering [5–8]. Although there are multiple mechanisms to promote tissue regeneration, like enhancing the proportion of transplanted MSCs that differentiate to the recipient cells and increasing the releasing of paracrine factors, the host’s immune response during the procedure of regeneration in skeletal muscle still remains to be explored. In this study, we found that MSCs and ECM scaffold promoted skeletal muscle regeneration by synergistically regulating macrophage polarization toward the M2 phenotype, which makes the compound containing MSCs and ECM a promising strategy for repairing VML.

As we all know, immune cells play an important role in tissue repair. During the tissue healing process, immune cells residing in the wound site control inflammation and promote tissue repair [46]. The macrophage is one of the most important functional cells in the immune system. In response to signals derived from the injury, the macrophage undergoes reprogramming which leads to the functional phenotypes. Depending on cytokines, such as IL-4, released from Th2 lymphocytes, expression levels of enzymes including Arg-1 and Fizz are upregulated, leading to the M2 phenotype which can relieve the inflammation and promote tissue regeneration [47, 48].

The immunomodulatory capacity of MSCs has been recognized as a significant principle for MSC-mediated therapy not only in autoimmune diseases but also in regenerative medicine [49]. According to previous studies, researchers found that there indeed exists a small amount of MSCs differentiating into different kinds of somatic cells like muscle cells, cartilage cells and cardiomyocytes using colocalization of immunofluorescence staining or species-specific qPCR [50–52]. However, with progress, researchers gradually came to realize that most of the implanted cells would die in a short period of time, and the result of regeneration is not mainly due to the rare MSC differentiation. MSCs are involved in tissue repair through the paracrine and immunomodulation effect [53, 54]. MSCs exert their immunomodulatory properties by regulating the function of both innate and adaptive immune cells through a mechanism involving both direct cell–cell contact and/or soluble factors [55–58]. A range of immune cells can be modulated by MSCs, including dendritic cells, natural killer cells, lymphocytes and macrophages. The inflammatory response is a crucial component of tissue regeneration, as evidenced by severely delayed repair following in-vivo macrophage ablation [29] and MSCs can directly modulate macrophages and their downstream functions [59, 60].

The ECM scaffold implantation can promote perivascular stem cell mobilization, increase the presence of neurogenic progenitor cells and is associated with myotube formation [61]. Recently, it has been reported that the ECM scaffold guided IL-4-dependent macrophage polarization through an mTOR/Rictor-dependent T-helper 2 pathway, which induced a proregenerative response, and was critical for functional muscle recovery [33]. These studies indicated that bioscaffolds can promote tissue regeneration by regulating the immune microenvironment alone. Here, we confirmed that not only MSCs but also ECM have the ability to suppress the secretion of inflammatory cytokines like TNF-α and IL-1β, and to enhance the secretion of anti-inflammatory cytokines like IL-4 and IL-10. More importantly, the compound containing MSCs and ECM scaffold can significantly control the level of inflammation, which may provide a reparative environment to activate the satellite cell for new muscle fiber formation. Meanwhile, MSCs and ECM scaffold can influence each other in wound healing [22]. This evidence suggests that ECM scaffold can improve MSC function by promoting the attachment and enhancing growth factor secretion. At the same time, MSCs may enhance the inducing effect of ECM during the tissue regeneration procedure.

Although we revealed that MSCs and ECM scaffold can have a synergistic effect on regulating macrophage polarization which is not just an additive effect, but the specific mechanism—especially the interaction between MSCs, ECM scaffold and macrophages—is unknown. Furthermore, the interactions between the ECM secreted by MSCs and decellularized ECM scaffold remain to be studied. The ECM secreted by MSCs and the decellularized ECM play important but different roles in tissue regeneration. Recent studies showed that the ECM secreted by MSCs could be the bridge to connect the cells and it can make MSCs easier to stay in place where they need to function. In addition, they can create the microenvironment to maintain homeostasis and they also can enhance the survival capacity of MSCs effectively [62, 63]. Meanwhile, the decellularized ECM mainly focuses on the recruitment of endogenous stem cells and immune cells to rebuild the destroyed microenvironment [33, 64]. Therefore, it is necessary to further explore the detailed molecular and cellular mechanisms of the different types of ECM to promote skeletal muscle regeneration. Also, it remains to be explored whether the combination of MSCs and ECM scaffold exerts a synergistic effect on satellite cell activation.

## Conclusions

In summary, the present study shows that when transplanted in a rat model of VML injury, MSCs and decellularized ECM scaffold had a synergistic effect on promoting skeletal muscle regeneration. The potential mechanism is that they change the default response to injury and facilitate a constructive remodeling outcome. This response is achieved by rapid and predominant macrophage polarization to the M2 phenotype. This synergistic immunoregulation effect of MSCs and ECM scaffold may provide an important regeneration mechanism regarding tissue engineering therapy for VML injury in the future.

## Additional file

Additional file 1: Shows construction of compound and TA muscle surgical procedure. (**A**) Heart decellularized extracellular matrix powder coated by one layer of cell aggregate to form compound. White arrows represent edge of cell aggregate. White dashed arrows represent compound. (**B–J**) About 20% of TA muscle excised during procedure to form irrecoverable VML injury. (TIFF 4627 kb)

## Abbreviations

ALP: Alkaline phosphatase
ANOVA: Analysis of variance
CFU-F: Fibroblastic colony-forming unit
COL: Collagen
ECM: Extracellular matrix
EDTA: Ethylenediamine tetraacetic acid
ES: Electric stimulation
FBS: Fetal calf serum
GAG: Glycosaminoglycan
H&E: Hematoxylin and eosin
IL-10: Interleukin-10
IL-13: Interleukin-13
IL-1β: Interleukin-1beta
IL-4: Interleukin-4
IL-6: Interleukin-6
iNOS: Inducible nitric oxide synthase
MSC: Mesenchymal stem cell
PBS: Phosphate buffer saline
SDS: Sodium dodecyl sulfate
SEM: Scanning electron microscopy
TA: Tibialis anterior
TNF-α: Tumor necrosis factor alpha
VML: Volumetric muscle loss
α-MEM: Minimum essential medium alpha
