# Supplementary figures and images for: Mesenchymal stem cells and extracellular matrix scaffold promote muscle regeneration by synergistically regulating macrophage polarization toward the M2 phenotype
Source: Stem Cell Res Ther. 2018 Apr 3;9:88. doi: 10.1186/s13287-018-0821-5 (PMC5883419; doi:10.1186/s13287-018-0821-5)

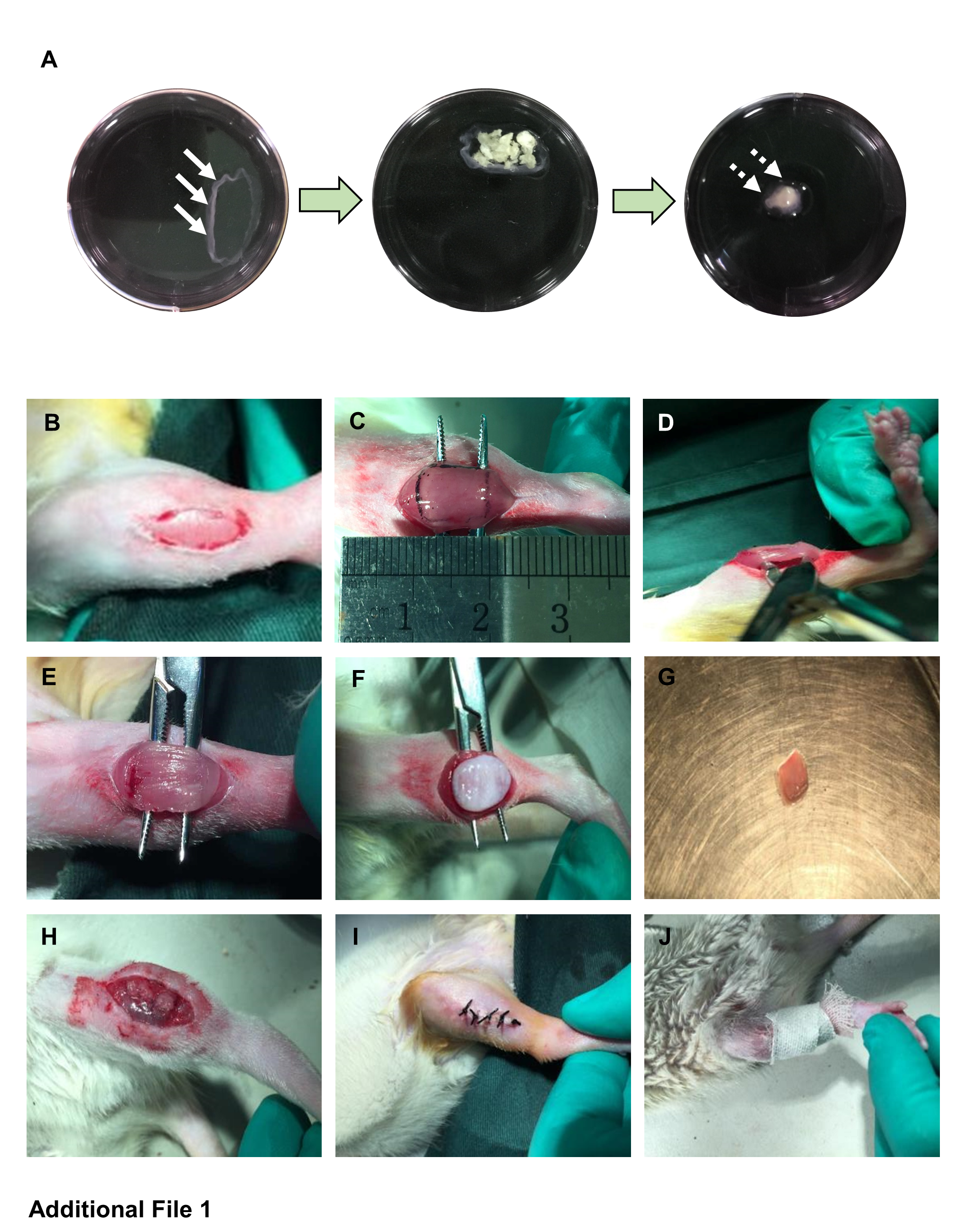

Supplement: Supplementary file 1 — Shows construction of compound and TA muscle surgical procedure. (A) Heart decellularized extracellular matrix powder coated by one layer of cell aggregate to form compound. White arrows represent edge of cell aggregate. White dashed arrows represent compound. (B–J) About 20% of TA muscle excised during procedure to form irrecoverable VML injury. (TIFF 4627 kb) [file 13287_2018_821_MOESM1_ESM.tif]
